# A Single-Center Retrospective Study of the Impact of Thyroid Cancer on the Malignant Risk of Contralateral TI-RADS 3 and 4 Nodules

**DOI:** 10.1155/2021/5538395

**Published:** 2021-10-07

**Authors:** Huan Liu, Jun Jin, Qiao Chen, Zhongmin Li

**Affiliations:** ^1^Department of General Surgery, Liaoyang Central Hospital of China Medical University, Liaoyang 111000, China; ^2^Department of Endocrinology, Liaoyang Central Hospital of China Medical University, Liaoyang 111000, China

## Abstract

**Background:**

The incidence of thyroid nodules increases in the general population. Similarly, we have also seen a dramatic increase in the number of thyroid surgeries. However, the mortality rate of thyroid cancer remained stable or even decreased. The purpose of our study was to investigate whether thyroid cancer affects the malignant risk of the contralateral TI-RADS 3 and 4 nodules.

**Methods:**

We conducted a retrospective cohort study in our institution for all thyroid procedures due to nodules from December 2018 to December 2019. All eligible patients were divided into the experimental group (bilateral nodules) and the control group (unilateral nodules) to assess whether the proportion of malignant nodules was different between the two groups. Multivariate logistic regression analysis was used to control potential confounding factors to investigate whether their differences were statistically significant.

**Results:**

A total of 330 patients underwent thyroid surgery, of whom 137 were eligible, including 84 in the experimental group and 53 in the control group. The proportion of malignant nodules was significantly different between the experimental group and the control group (29.8% versus 58.5%, unadjusted OR 0.30, 95% CI: 0.17–0.82, *p*=0.001). However, after controlling for potential confounding factors, including age (*p*=0.004), gender (*p*=0.775), and TI-RADS classification (*p* ≤ 0.001), we found that the difference was not significant (adjusted OR 1.08, 95% CI: 0.39–3.01, *p*=0.886).

**Conclusion:**

There is no evidence that thyroid cancer affects the malignant risk of the contralateral TI-RADS 3 and 4 nodules. This study has been registered with the Chinese Clinical Trial Registry (clinical trial registration number: ChiCTR2000038611, registration time: September 26, 2020).

## 1. Introduction

Thyroid nodules are defined as discrete lesions within the thyroid, which are radiologically different from the surrounding thyroid parenchyma [1]. It is extremely common in the general population, reaching 4%–7% and 30%–67% in palpation and Imaging examination, respectively [2]. Currently, more thyroid nodules are identified by occasional imaging studies, rather than thyroid-related symptoms or palpation. Moreover, these occasional thyroid nodules are asymptomatic and small [3]. The incidence of occult thyroid nodules in the general population is as high as 68% [2], and many imaging studies are beneficial to identify these thyroid nodules. Just as previous studies have shown the incidence of thyroid nodules in ultrasound, computed tomography (CT), or magnetic resonance imaging (MRI), 18-fluorodeoxyglucose positron emission tomography (PET) is 65%, 15%, and 1%–2%, respectively [4]. Among them, ultrasound is the most common imaging examination for thyroid nodules, and it is recommended by the British, American, and European Thyroid Association (BTA, ATA, and ETA, respectively) as the preferred imaging method to evaluate thyroid nodules [1, 5, 6]. Thyroid ultrasound can accurately identify malignant thyroid nodules and may even be better than fine-needle aspiration biopsy (FNAB) [7, 8].

Thyroid nodules include benign and malignant ones, and the incidence of malignant nodules varies from 4% to 12%, including incidental thyroid nodules [7, 9–16]. Thyroid cancer currently ranks fifth among female cancers, but it is estimated that thyroid cancer will rank second and ninth among female and male cancers by 2030 [17]. However, not all thyroid nodules require surgery or even biopsy. More than 90% of thyroid nodules are of no clinical significance because there are no malignant ultrasound features or pathologically proven benign [18]. The emergence of the Thyroid Imaging Report and Data System (TI-RADS) [19], developed by the American College of Radiology (ACR), allows clinicians to stratify the risk of thyroid nodules and take appropriate management strategies. This is conducive to timely treatment of clinically significant thyroid cancer and can also reduce costs and risks due to biopsy and surgery for benign thyroid lesions and indolent thyroid cancer. Previous studies reported that the malignant risk in TI-RADS1-5 is significantly different, which are <2%, <2%, 5%, 5%–20%, and ≥20%, respectively.

We have noted an interesting phenomenon that the incidence of thyroid nodules is increasing; however, the absolute mortality of thyroid cancer has remained stable or even decreased. This may be caused by the current more aggressive management strategy for thyroid nodules, which is not only reflected in the number of procedures, but also reflected in its scope (preferably total thyroidectomy), even for the low-to-medium risk thyroid cancer [20]. Based on the 2017 ACR Thyroid Guidelines and many subsequent studies including prospective and retrospective clinical trials, there is almost no controversy regarding the treatment of TI-RADS 1 and 2 nodules because almost all are benign. Similarly, the treatment of TI-RADS 5 nodules is also not controversial due to the extremely high risk of malignancy. However, the treatment of TI-RADS 3 and 4 is still controversial. Moreover, thyroid nodules are characterized by bilateral ones, which makes the surgeon confused about whether bilateral thyroid requires surgery and what its scope is. Therefore, this leads to more complicated and confusing treatments for bilateral nodules, especially TI-RADS 3 and 4, which vary based on surgeons' preferences and institutional experience. Because of the relatively independent anatomical characteristics of bilateral thyroid, we consider whether thyroid cancer will affect the malignant risk of the contralateral thyroid nodule, and whether this may also be an influencing factor in the treatment of bilateral thyroid nodules. However, there are few related studies.

Therefore, we regarded bilateral thyroid glands as two independent parts to investigate whether thyroid cancer affects the malignant risk of the contralateral TI-RADS 3 and 4 nodules.

## 2. Materials and Methods

### 2.1. Population and Data Collection

This study was a single-center retrospective cohort study. We reviewed patients with thyroid nodules in our hospital from December 1, 2018, to December 31, 2019, through the electronic case system. The data included demographic characteristics (including age and gender), medical history, thyroid ultrasound results, surgical procedures, and postoperative pathology. All thyroid ultrasound examinations were completed by 5 experienced sonographers within 3 days after admission or in the clinic and reviewed by chief physicians with more than 25 years of experience. The thyroid ultrasound reports included the number, size, location, grade of the thyroid nodules, and cervical lymph nodes. The thyroid nodule grading was based on the 2017 ACR Thyroid Imaging Report and Data System (TI-RADS) guidelines.

### 2.2. Research Objects and Groups

All eligible patients were divided into the experimental group and the control group as follows. To facilitate the subsequent statistical analysis, we had a special definition for bilateral thyroid nodules. For bilateral thyroid nodules, the side with higher TI-RADS grade or more advanced pathology was the b-side, while the contralateral was the a-side. For example, the side with anaplastic or medullary cancer was the b-side, and the contralateral papillary carcinoma was the a-side. When both thyroid cancers were papillary cancers, the side with higher TI-RADS grade was the b-side. In our study, statistical analysis was performed on the a-side of the experimental group and the control group.

The inclusion criteria of the experimental group were as follows: (1) bilateral thyroid nodules, (2) bilateral thyroid nodules that were surgically removed, and (3) bilateral thyroid nodules that had postoperative pathology. Exclusion criteria of the experimental group were as follows: (1) bilateral benign nodules, (2) history of malignant tumors in other tissues or organs (except thyroid), (3) history of thyroid procedures due to thyroid nodules or other thyroid diseases, and (4) the a-side thyroid with TI-RADS 1, 2, and 5 nodules.

The inclusion criteria of the control group were as follows: (1) unilateral thyroid nodules, (2) unilateral nodules that were surgically removed, and (3) unilateral thyroid nodules that had postoperative pathology. Exclusion criteria of the control group were as follows: (1) history of malignant tumors in other tissues or organs (except thyroid), (2) history of thyroid procedures due to thyroid nodules or other thyroid diseases, and (3) patients with TI-RADS 1, 2, and 5 nodules.

### 2.3. Statistics

In our study, continuous variables were described by mean ± standard deviation (SD), which were statistically analyzed by Student's *t*-test or Mann–Whitney *U* test according to its distribution. Categorical variables were described by frequency (percentage), which were statistically analyzed by Chi-square tests or Fisher's exact tests as appropriate. We evaluated the unadjusted odds ratio (OR) based on the proportion of malignant tumors in the experimental group and the control group, which was described as unadjusted OR, 95% CI. In order to eliminate confounding factors, including age, gender, and TI-RADS classification, we used multivariate logistic regression analysis to evaluate the adjusted odds ratio, expressed as adjusted OR, 95% CI. *p* value ≤ 0.05 was considered statistically significant. All statistics were performed with SPSS version 25.0.

## 3. Results

**Figure 1:**
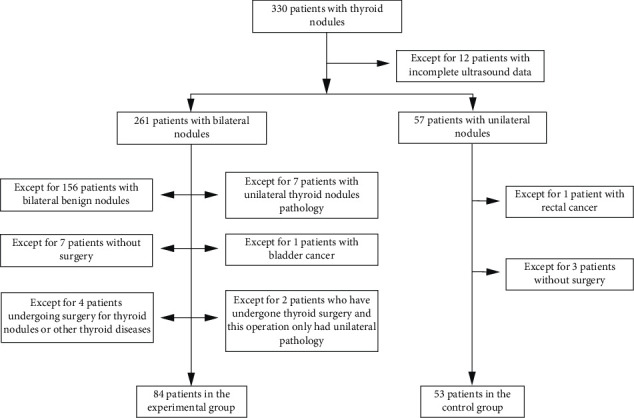
Flowchart of review and grouping of the study.

The review and grouping of the study are shown in Figure 1. Our institution, a tertiary regional medical center, performed 330 thyroid procedures due to thyroid nodules within 1 year. A total of 137 eligible patients were divided into the experimental group (*n* = 84) and the control group (*n* = 53).

After excluding 12 patients with incomplete information, the distribution of TI-RADS grades for 261 bilateral nodules and 57 unilateral nodules is shown in Table 1. We saw that bilateral thyroid nodules were more common (81.5% versus 18.5%). Moreover, most thyroid nodules were TI-RADS 3 and 4 ones, and TI-RADS 2 and 5 nodules were extremely rare, especially in unilateral thyroid nodules.

The demographic characteristics of the experimental group and the control group are shown in Table 2. We saw that femal were more common in both the experimental group and the control group; however, there was a statistical difference between the two groups (*p*=0.008). The average age in the experimental group and the control group was 50.8 ± 11.0 years and 47.7 ± 9.6 years, respectively, and there was no difference between the two groups (*p*=0.246).

The ratio of malignant nodules in the two groups is shown in Table 3. There was a significant difference in the proportion of malignant nodules between the a-side of the experimental group and the control group (29.8% versus 58.5%, unadjusted OR 0.30, 95%CI: 0.17–0.82, *p*=0.001). It showed that thyroid cancer does not increase the malignant risk of the contralateral TI-RADS 3 and 4 nodules; on the contrary, its risk was lower. However, we used multivariate logistic analysis to investigate whether age, gender, and TI-RADS grade, respectively, affect the proportion of malignant nodules in the two groups and found that age (*p*=0.004) and TI-RADS classification (*p* ≤ 0.001) were statistically significant, while gender was not statistically significant (*p*=0.775). Therefore, after controlling for potential confounding factors, such as age (*p*=0.004), gender (*p*=0.775), and TI-RADS classification (*p* ≤ 0.001), we found that thyroid cancer does not affect the malignant risk of the contralateral TI-RADS 3 and 4 nodules (adjusted OR 1.08, 95% CI: 0.39–3.01, *p*=0.886).

The characteristics and surgical procedures of the a-side of the experimental group and the control group are shown in Table 4.

The distribution of TI-RADS classification on the a-side of the experimental group and the control group were significantly different (*p* ≤ 0.001). The nodules on the a-side of the experimental group were more TI-RADS3 nodules; on the contrary, the control group was TI-RADS4 nodules. In the a-side of the experimental group and the control group, all malignant nodules were papillary thyroid cancer, and the proportion of micropapillary cancer was not different between the two groups (*p*=0.282). We knew that the b-side in the experimental group was thyroid cancer, and the a-side surgical procedures varied with its pathology.

When the nodules on the a-side of the experimental group and the control group were malignant, there was no difference in surgical procedures between the two groups (92.0% versus 96.8%, *p*=0.581). However, when it was benign, there was a significant statistical difference between the two groups (50.8% versus 13.6%, *p*=0.002), and the a-side of the experimental group was more inclined to unilateral lobectomy.

The pathological distribution of b-side thyroid cancer in the experimental group is shown in Table 5. It included 82 (97.6%) papillary carcinomas, 1 (1.2%) medullary carcinoma, and 1 (1.2%) anaplastic carcinoma. We saw that the vast majority of them were low-to-medium risk papillary cancers, and the more indolent micropapillary cancers were relatively high.

## 4. Discussion

Thyroid nodules are an extremely common disease, with a high incidence in the general population. In China, more and more thyroid procedures are due to physical examinations or incidentally identified thyroid nodules, which is consistent with studies from other countries and regions. In order to take appropriate treatment for thyroid nodules, the ACR developed TI-RADS in 2017. However, the treatment of TI-RADS 3 and 4 nodules is controversial, especially its procedures and scope. At present, thyroid nodules encountered in clinical practice are basically TI-RADS 3 and 4 nodules. In our study, its proportion reached 100% and 97.62% in unilateral and bilateral nodules, respectively. Thyroid nodules are usually characterized by bilateral nodules, which leads to more complicated and confusing surgical procedures for TI-RADS 3 and 4 nodules [20]. The procedures for patients with both benign and malignant nodules are clear. However, patients with benign on one side and malignant on the contralateral are controversial. Among them, there are some patients whose one side has been pathologically confirmed as malignant, but the contralateral TI-RADS 3 or 4 nodules may not be palpable during the operation. Whether this side requires procedure, if necessary, what is its scope, there is no consensus on these issues. Similarly, this controversy also exists in those patients whose nodules on one side have been confirmed as low-to-medium risk papillary carcinoma and contralateral micropapillary carcinoma.

A systematic review including 14 studies at a moderate risk of bias found the odds ratio for thyroid cancer to be lower in patients with a multinodular goiter than in those with single nodule [21]. In our study, we found that thyroid cancer significantly reduced the malignant risk of the contralateral TI-RADS 3 and 4 nodules (29.8% versus 58.5%, unadjusted OR 0.30, 95% CI: 0.17–0.82, *p*=0.001). However, we used multivariate logistic regression analysis to control potential confounding factors, including age (*p*=0.004), gender (*p*=0.775), and TI-RADS classification (*p* ≤ 0.001), and found that the difference did not reach statistical difference (adjusted OR 1.08, 95% CI: 0.39–3.01, *p*=0.886).

We know that more than 90% have no clinical significance, especially nodules smaller than 1 cm. Therefore, not all thyroid nodules require surgery [18, 22–27]. Benign thyroid nodules only require regular ultrasound follow-up unless the nodules are large (>4 cm) or based on clinical considerations or cause compression or structural symptoms [22, 28]. For thyroid cancer, the proportion of papillary cancer is extremely high, and other thyroid cancers are rare. In our study, there were 1 medullary carcinoma and 1 anaplastic carcinoma. Moreover, papillary thyroid cancers have a favorable prognosis, with mortality rates of 1% to 2% at 20 years [29]. It is generally perceived as low-risk thyroid cancer [30] and is not associated with well-recognized predictors of mortality [31, 32]. The American Thyroid Association (ATA) guidelines recommend lobectomy for low-risk nodules and thyroidectomy (including total thyroidectomy and subtotal thy*r*oidectomy) or lobectomy for intermediate-risk nodules [1]. Moreover, many studies have demonstrated that overall survival and disease-free survival are not negatively impacted by lobectomy compared with thyroidectomy [33–39]. However, for those patients whose one side nodules have been proven to be low-to-medium risk papillary cancer and the contralateral nodules are micropapillary cancer, or even benign ones, surgeons in China usually prefer total thyroidectomy. In our study, we did not find that thyroid cancer increases the risk of malignancy of the contralateral TI-RADS 3 and 4 nodules. However, in our study, we saw that the a-side nodules were malignant, even though 72% of them were micropapillary carcinomas and 92% of them had undergone lobectomy. Similarly, even if the a-side nodules were benign, the a-side of the experimental group was more inclined to lobectomy (50.8% versus 13.6%, *p*=0.002). To a certain extent, this indicates a more active management strategy, which is common not only in China but also in other regions.

The increase in the incidence of thyroid nodules can be seen worldwide [40]. In the US, a retrospective population-based evaluation of patients with thyroid cancer found that the incidence increased from 3.6/100000 in 1973 to 8.7/100000 in 2002, a 2.4-fold increase [41]; similarly, it increased 15 times in South Korea between 1993 and 2011 [42, 43]. However, the mortality rate of thyroid cancer remained stable or even declined [41–46]. At the same time, we have also noticed that the number of thyroid procedures increased dramatically, reaching 2–4 times [44, 45]. Another study showed that it increased from 99,613 to 130,216 per year in the United States from 2006 to 2011, with an average annual growth rate of 12% [47]. Moreover, the number of thyroid procedures has increased disproportionately with its morbidity and mortality. This evidence also indirectly indicated that thyroid nodules tend to be more aggressive management strategies in clinical practice. Similarly, many studies have confirmed that more resources were used for the diagnosis, treatment, and follow-up of those thyroid cancers, which may not affect the life of the patient or even have no symptoms [48, 49]. A large observational study based on the SEER database found that after controlling tumor size, for those patients with low-risk tumors, aggressive surgical treatment has no benefit in survival [36]. Several studies have indirectly demonstrated the current status of overtreatment of thyroid nodules [21, 45, 47–50].

When it comes to procedure, postoperative complications are an inevitable issue. A study showed that there was recurrent laryngeal nerve injury (2.5%, rare on both sides), hypocalcemia (8.1%) after total thyroidectomy [51]. Do more aggressive procedures for thyroid nodules increase the risk of postoperative complications? A study showed that there is no correlation between surgical procedures and the incidence of postoperative hypocalcemia [52]. However, another study found that total thyroidectomy is more prone to postoperative complications than subtotal thyroidectomy, including permanent nerve damage (7.0% versus 1.3%, *p* < 0.005) and temporary nerve damage (8.6% versus 2.2%, *p* < 0.005) and transient hypoparathyroidism (18.0% versus 2.1%, *p* < 0.005) [33]. Moreover, lobectomy can provide histological diagnosis and tumor removal with a lower risk of complications [18]. In our study, we did not find that thyroid cancer increases the risk of malignancy of the contralateral nodule. Therefore, the current more aggressive treatment strategy for thyroid nodules may not be a good choice for patients and society. For those patients with low-to-medium risk papillary cancer on one side, if the contralateral TI-RADS 3 and 4 nodules cannot be palpated during the operation, they can be followed up instead of lobectomy [20]. If the contralateral side is micropapillary carcinoma, or benign, partial lobectomy can be performed, which can retain enough thyroid tissue to meet normal physiological needs without affecting the prognosis of the patient while reducing the possibility of postoperative complications [20]. Moreover, in recent years, the epidemic has led to inadequate medical funding in various countries. These issues are worthy of our understanding of the current status of the treatment of thyroid nodules, and further research on its management strategies.

In our study, we found that thyroid cancer does not affect the malignant risk of the contralateral TI-RADS 3 and 4 nodules. However, there are still many limitations to be improved. First, our study is a single-center retrospective study with its own limitations, and we hope that it can be carried out in multicenter and larger-scale cases in the future. Second, we only investigated the effect of thyroid cancer on the contralateral TI-RADS 3 and 4 nodules, while TI-RADS 5 nodules did not. This effect may be more significant in TI-RADS 5 nodules. Similarly, because almost all thyroid cancers in our study are papillary cancers, whether this effect of other thyroid cancers may be more significant remains to be studied in the future. Third, our research is lacking in terms of postoperative complications and survival rates. Moreover, there are few studies on the correlation between the scope of surgery and postoperative complications and survival rate. Therefore, we need to do further research on comprehensive management strategies to find a balance between the treatment effect, the risk of postoperative complications, and the economy. It enables timely and appropriate treatment of clinically significant thyroid nodules and does not increase the patient's physical, psychological, and economic burdens and cause a waste of medical resources. At the same time, other conservative treatment methods, such as thermal ablation, laser ablation, radiofrequency ablation, and microwave ablation, also provide alternative options.

Finally, the issue of fine-needle aspiration biopsy (FNAB) of thyroid nodules is specifically explained. Many thyroid treatment guidelines recommend FNAB before surgery, but FNAB has a high proportion of insufficient biopsy specimens and indeterminate pathological results. For those patients with insufficient biopsy specimens or indeterminate pathological results, FNAB may need to be performed again. However, some patients may not have clear pathological results after repeated FNAB. In our study, surgeons advised all patients with thyroid nodules to perform FNAB but fully informed them of the pros and cons, and almost all patients refused to perform FNAB. Because of the improvement of surgical techniques, anesthesia, and nursing care, the length of hospital stay has been shortened, which is another reason.

## 5. Conclusions

In our study, there is no evidence that thyroid cancer affects the malignant risk of the contralateral TI-RADS 3 and 4 nodules.

## Figures and Tables

**Table 1:** Distribution of thyroid nodule TI-RADS classification.

	Unilateral nodules *n* (%)	Bilateral nodules *n* (%)
2/2	0 (0)	0 (0)
2/3	—	1 (0.4)
2/4	—	3 (1.2)
2/5	—	0 (0)
3/3	17 (29.8)	122 (46.7)
3/4	—	102 (39.1)
3/5	—	12 (4.6)
4/4	40 (70.2)	19 (7.3)
4/5	—	0 (0)
5/5	0 (0)	2 (0.7)
Total	57 (18.5)	261 (81.5)

**Table 2:** Demographic characteristics of the experimental group and the control group.

	Experimental group	Control group	*p* value
*Gender*			
Male *n* (%)	10 (11.9)	16 (30.2)	0.008
Female *n* (%)	74 (88.1)	37 (69.8)	
Age, years, (mean ± SD)	50.8 ± 11.0	47.7 ± 9.6	0.246

SD: standard deviation.

**Table 3:** The ratio of malignant tumors on the a-side of the experimental group and the control group.

	Experimental group (*N* = 84)	Control group (*N* = 53)	Unadjusted OR (95% CI)	*p* value	Adjusted OR (95% CI)	*p* value
Benign *n* (%)	59 (70.2)	22 (41.5)	0.30 (0.17, 0.82)	0.001	1.08 (0.39, 3.01)	0.886
Malignant *n* (%)	25 (29.8)	31 (58.5)				

OR: odds ratio; adjusted OR: control for confounding factors, including age (*p*=0.004), gender (*p*=0.775), and TI-RADS classification (*p* ≤ 0.001).

**Table 4:** Characteristics and surgical procedures of the a-side of the experimental group and the control group.

	Experimental group	Control group	*p* value
TI-RADS classification			
TI-RADS 3 *n* (%)	65 (77.4)	15 (28.3)	≤0.001
TI-RADS 4 *n* (%)	19 (22.6)	38 (71.7)	
Pathology			
Micropapillary *n* (%)	18 (72.0)	26 (83.9)	0.282
Papillary *n* (%)	7 (28.0)	5 (16.1)	
Surgical procedures			
Malignant^*∗*^			
UL^□^*n*(%)	23 (92.0)	30 (96.8)	0.581
UPL^∆^*n*(%)	2 (8.0)	1 (3.2)	
Benign^*∗*^			
UL^□^*n*(%)	30 (50.8)	3 (13.6)	0.002
UPL^∆^*n*(%)	29 (49.2)	19 (86.4)	

UL^□^: unilateral lobectomy; UPL^∆^: unilateral partial lobectomy; malignant^*∗*^and benign^*∗*^: pathology of the a-side of the experimental group and the control group.

**Table 5:** Pathological types of b-side thyroid cancer in the experimental group.

	Papillary thyroid cancer		Medullary	Follicular	Anaplastic
	Yes^*∗*^	no			
Experimental group *n* (%)	38 (45.2)	44 (52.4)	1 (1.2)	0 (0.0)	1 (1.2)

^*∗*^Thyroid micropapillary carcinoma.

## Data Availability

The data used during the current research are available from the corresponding author on reasonable request.
